# Bilateral Talus and Navicular Fractures accompanied with Unilateral Calcaneal Fracture: A Case Report

**DOI:** 10.5704/MOJ.1807.009

**Published:** 2018-07

**Authors:** G Bulut, I Colak, G Mik, Z Kilic, Z Tasdemir

**Affiliations:** Department of Orthopaedics, Dr Lutfi Kirdar Kartal Training and Research Hospital, Istanbul, Turkey; ^*^Department of Orthopaedics, Istanbul Surgery Hospital, Istanbul, Turkey

**Keywords:** trauma, fractures, sprains and strains, talar fractures, multiple isolated tarsal fractures in foot

## Abstract

An 18-year old male patient, with a history of paragliding accident, sustaining a coronal shear fracture of the body of the talus, an anterior process fracture of the calcaneus extending to the calcaneocuboid joint and a nondisplaced navicular body fracture at the right foot and a displaced fracture of the navicular body accompanied with posteromedial process fracture of the talus at the left side was referred to our emergency clinic. For the right foot, the coronal plane fracture of the talar body was anatomically reduced and fixed with screws. For the left foot, screw fixation was performed through the lateral aspect to fix the large posteromedial fragment. Small bone fragments were removed from the left navicular fracture, and the main fragments were also fixed with screw. The talo-navicular joint was stabilised with a Kirschner wire. At 36 months follow-up, bilateral foot and ankle functions were satisfactory, Maryland scores of the right and left foot were 85 (good) and 90 (excellent), respectively, and the patient regained his full activity level by the 5th month postoperatively. With reference to the number and types of fractures in this one patient, we present a standard protocol for treatment of isolated talus, navicular and calcaneal fractures presenting together in a single foot injury.

## Introduction

The incidence of fractures of the talus ranges from 0.1 to 0.85% of all fractures. Majority of the talus fractures are marginal and avulsion types, and the next common are neck fractures. Talar body fractures comprise 13 to 23% of talus fractures. Avulsion fractures of the navicular bone are the most common type of navicular fracture. Fractures of the tarsal navicular body are less common but occur with higher energy trauma and are potentially much more serious than avulsion fractures^1^. Combined ipsilateral talar and calcaneal fractures is an unusual injury pattern and has been rarely reported in wide series^2^. Isolated injuries to either of these bones has been frequently reported in trauma series and specific treatment protocols with various complication rates^1,3^. We present an uncommon injury including rare types of fractures of talus, navicula and calcaneus, with a review of the literature.

## Case Report

An 18-year old male patient was referred to our emergency clinic with injuries sustained in a paragliding accident. During takeoff from the slope with a parachute, and after achieving an altitude of 10-15 meters, the patient stated that he had dropped on his feet due to loosening of the security ties.

**Fig. 1:**
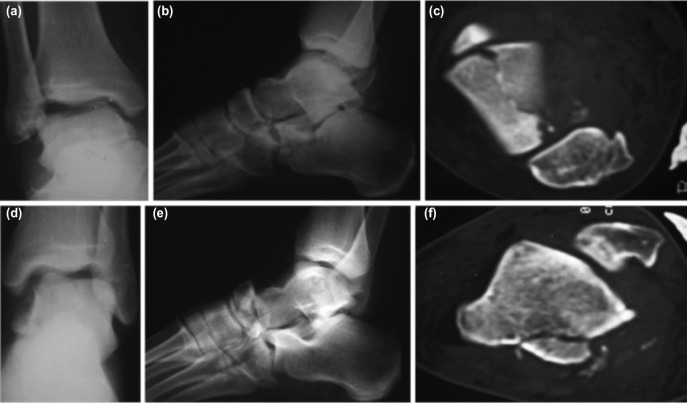
Pre-operative radio-imaging of both feet (a) anterior-posterior radiograph of right ankle showing coronal shear fracture of the body of the talus. (b) lateral radiographs showing anterior process fracture of the calcaneus extending to the calcaneocuboid joint and nondisplaced navicular body fracture. (c) CT scan of the right foot and ankle, showing coronal shear fracture of the body of the talus. (d) anterior-posterior radiograph of left ankle showing subluxation of talus with no obvious fracture. (e) lateral radiographs showing displaced fracture of the navicular body with talo-navicular subluxation. (f) CT scan of the left foot and ankle showing fracture of posteromedial process fracture of the talus.

Physical examination revealed limitation of movement, ecchymosis, and edema over both feet and ankles. Pain and tenderness were especially localised anteriorly on the right ankle and dorsally over the calcaneocuboid joint on the right foot, and anteromedially on the left foot and around the left medial malleolus. There was no open wound and neurovascular status was intact in both feet. Antero-posterior (AP) and lateral (LAT) radiographs of the foot and ankle revealed coronal shear fracture of the body of the talus, an anterior process fracture of the calcaneus extending to the calcaneocuboid joint and a nondisplaced navicular body fracture of the right foot (Fig. 1a-c) and a displaced fracture of the navicular body accompanied with posteromedial process fracture of the talus on the left foot (Fig. 1d-f).

To determine the exact localisation of the fragments and the degree of fracture displacement more accurately, a computerised tomography (CT) scan was performed. This revealed the coronal shear fracture of the talar body and the navicular fracture displaced 3mm and 1mm respectively, while the anterior process fracture of the calcaneus was minimally displaced on the right foot (Fig. 1a-c). On the left foot, the posteromedial process fracture of the talus was displaced approximately 3mm, and revealed fragmentation, and that the navicular fracture consisted of three fragments and was dorsally displaced (Fig. 1d-f).

**Fig. 2:**
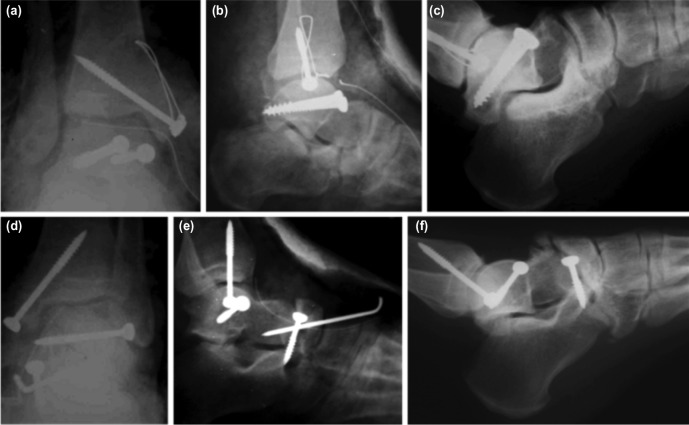
Post-operative radio-imaging of both feet (a) anterior-posterior and (b) lateral radiographs of right ankle showing anatomically reduced right talar fracture with screw fixation. The medial malleolus osteotomy was fixed with one screw and figure-eight wire looping (c) lateral radiographs of the same foot at 36 months follow-up. (d) anterior-posterior. (e) lateral radiographs of the left foot showing anatomic reduction and screw fixation of the left talus posteromedial process fracture, screw fixation of left navicular bone, and Kirschner wire fixation of left talo-navicular joint. (f) lateral radiographic of the same foot at 36 months follow-up.

The patient was operated on eight hours from the time of the trauma. Under general anaesthesia and pneumatic tourniquet, an anteromedial incision was made for the talar body fracture at the right side. Neurovascular structures were identified and protected, and an oblique osteotomy was performed on the medial malleolus. The talar body fracture was visualised. The vertical fracture line in the coronal plane was close to the posterior and displaced causing stepping-off on the talar dome but had no fragmentation. Following curettage and irrigation of the fracture site, it was anatomically reduced, and fixation was performed with two 4.5mm self-tapping screws from anterior to posterior to obtain compression. The medial malleolus osteotomy was fixed with one 4.5mm self-tapping screw and figure-eight wire looping. Fractures of the anterior process of the calcaneus and navicular body were managed non-surgically. The foot and leg were immobilised in plaster cast postoperatively (Fig. 2a-c).

An anteromedial incision was also used for the left foot. Following the identification and retraction of the neurovascular structures, an oblique medial malleolus osteotomy was carried out. Posteromedial process of the talus was explored. After anatomic reduction, a small lateral incision was made and a 4.5mm self-tapping screw was placed through the lateral aspect for fixation of the large posteromedial fragment. Extending the incision towards the navicular bone, the fracture line and talonavicular joint were explored. The small bone fragments at the fracture site were removed. After anatomic reduction, fixation was achieved utilising a 4.5mm self-tapping screw from the dorsal to the plantar aspect. The talonavicular joint was stabilised with a Kirschner wire from anterior to posterior, and it was left extruding on the skin. Medial malleolus osteotomy was fixed with a 4.5mm self-tapping screw. A short leg cast was applied postoperatively (Fig. 2d-f).

**Fig. 3:**
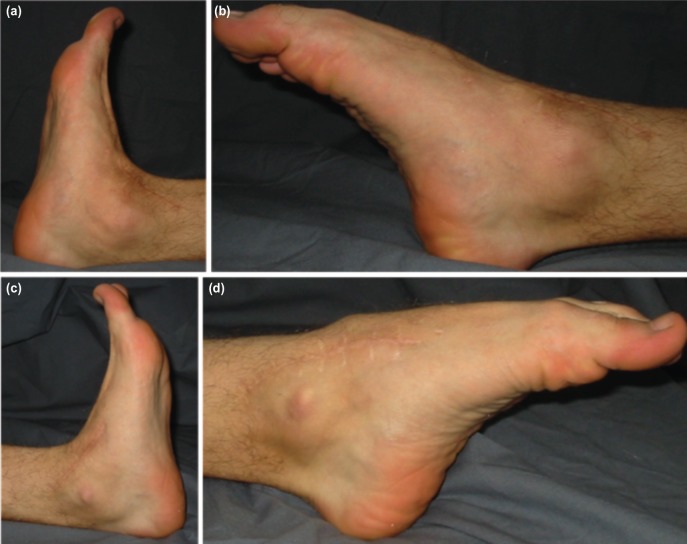
Photographs showing range of motion of both feet at 36 months follow-up. (a) dorsal flexion. (b) plantar flexion of the right foot. (c) dorsal flexion. (d) plantar flexion of the left foot.

Parenteral cefazolin sodium (3x1 gr/day) was administered for three days postoperatively. After six weeks of immobilisation, plaster casts were removed and the Kirschner wire stabilising the talonavicular joint was pulled out. Radiographic evaluation following a rehabilitation period of six weeks showed consolidation and union of all fractures without any loss in reduction in both feet, and full weight bearing was encouraged. At 36 months follow-up there was no evidence of avascular necrosis. All fractures had completely healed. There were minimal arthritic changes at the right subtalar and calcaneocuboid and left talonavicular joints. There was no complaint of pain and the range of motion of both feet and ankles were near full. The patient regained his full activity level at the 5th month postoperatively. Maryland scores of the right and left feet were 85 (good) and 90 (excellent), respectively (Fig. 3).

## Discussion

Midfoot fractures are rarely seen because of the geometrical configurations of the tarsal bones and strong ligaments holding them together. However, morbidity of those fractures is high. Rarely seen talus and navicula fractures can easily be overlooked during the initial examination^1,3^. Navicular fractures accompanied with a calcaneus anterior process fracture extending to the calcaneocuboid joint are reported in the literature^4^. Seybold *et al* investigated 950 patients with calcaneal fractures and 190 patients with talar fractures treated operatively over a 20-year period, and only 11 patients (1% of calcaneal and 6% of talar fractures) were identified with combined ipsilateral talar and calcaneal fractures^2^. Extra-articular calcaneal fractures are more common in this injury pattern, while there was no preference for either talar neck or talar body fractures^2^. In our case, there was a combined ipsilateral talar body fracture with the anterior process fracture of the calcaneus. Authors concluded that the combination of ipsilateral talar and calcaneal fractures represented severe injury pattern associated with significant clinical and radiographic morbidity and long-term sequel, especially subtalar arthritis was a common finding regardless of treatment^2^. Isolated posteromedial process fracture of the talus is also an extremely rare injury and only ten cases have been reported in the literature^5^.

Forty-seven percent of tarsal navicula fractures are avulsion fractures. Avulsion fractures are treated non-surgically even if they are displaced with avulsions caused by tibialis posterior tendon. Body fractures of the tarsal navicular bone are rare but serious fractures. They usually occur due to indirect axial loading. The direction and the amount of the force applied to the forefoot determine the pattern of the fracture and the degrees of displacement. Forces causing hyperdorsiflexion result in displacement of the navicular bone dorsally^1^. These fractures are usually classified as displaced or non-displaced fractures, while Sangeorzan *et al* divided displaced fractures into three subgroups^1^. According to this classification, the fracture of the left navicular bone in our case is a type II fracture. The fracture line extends from dorsolateral to medial plantar aspect and the large medial fragment subluxated dorsomedially. Reducing type II injuries can be difficult. The large medial fragment of the navicular bone must be reduced to inspect the talonavicular joint. Fixation of this type of fractures with a lag screw if comminution is present, stabilisation of cuneiforms by another screw and additionally fixation of the talonavicular joint by a Kirschner wire is recommended^1^. As described in the literature, we fixed the fracture with a 4.5mm self-tapping screw and stabilised the talonavicular joint with a Kirschner wire. Navicular fracture on the right foot was undisplaced and treated non-surgically.

The final lateral radiograph demonstrates that peritalar fracture-dislocation on the left side, was partially reduced, most obviously at the posterior facet of the subtalar joint. We assume that this was likely due to failure to restore the length of the medial column of the foot, which is shortened here due to the navicular fracture. A medial external fixator or distractor could have helped to achieve this intra-operatively, while also making navicular fixation easier, but we could not achieve this during the operation. However, the final clinical outcome was better than we had hoped. There were minimal arthritic changes at the left talonavicular joint and there was no pain. The patient regained his full activity level and Maryland score of the left foot was 90.

Posteromedial process fractures are the rarest types of talus fractures and a small number of cases were reported in the literature. This kind of fracture pattern of talus is often treated as soft tissue injury of the ankle and misdiagnosed. Therefore, CT scans of patients referring with ankle or foot sprains and having pain medial to the talus may be helpful for diagnose. Hyperdorsiflexion was present and it was assumed to be responsible for the fractures with a pronation injury which readily accompanied the mechanism. Additionally, there was a common opinion related with the avulsion injury of the posterior talotibial ligament as being the underlying reason^4,5^. Reduction of the fracture through medial malleolus osteotomy and fixation with bioabsorbable pins was recommended. We also performed a medial malleolus osteotomy for the treatment of the posteromedial process fracture in our case and, following open reduction, we carried out a fixation for the large posteromedial fragment with a 4.5mm self-tapping screw from lateral to medial aspect through a mini lateral incision. Thus, the head of the screw did not remain under the osteotomised medial malleolus. Various complications, mainly subtalar arthritis and avascular necrosis, have been reported after talus fractures^3^. Since early and anatomically reduction was performed, neither serious arthritis nor avascular necrosis was seen in our case.

Calcaneus anterior process fracture and the avulsion fracture of the navicula are the fracture patterns caused by acute dorsiflexion and valgus stress. This type of injury was classified as “lateral strain” sub-type in Main and Jowett’s classification for midtarsal fractures and subluxations and reported in 22 cases^4^. They recommended immobilisation or calcaneocuboid fusion options for the treatment of such fractures and pointed out that the treatment must be arranged according to the degree of the displacement in the calcaneocuboid joint^4^. Following CT scan evaluation, we preferred to immobilise this fracture by plaster cast.

The mechanism of injury for the coronal shear fractures of the talar body consist of hyperdorsiflexion with axial compression like talar neck fractures^3^. The recommended treatment method in such fractures is open reduction by medial malleolus osteotomy and fixation with lag screws unless comminution is present^3^. We also performed internal fixation of our patient’s right talar body fracture by using this method and had no complication.

As we could not identify a similar case in the literature, both in terms of number and types of fractures and injury mechanism, we considered hyperdorsiflexion mechanism of both feet with the axial loading in the foreground, resulting from a fall from a height onto an inclined surface and with forward motion during paragliding, was the determinant of the underlying injury. Although there are so many options for choosing the treatment regimen of this specific case, we chose to treat this combined foot injury by standard protocols for treatment of isolated talus, navicular and calcaneal fractures.

## Conflict of Interest

The authors declare no conflicts of interest.
